# Characterization of the epithelial sodium channel α subunit coding and non-coding transcripts and their corresponding mRNA expression levels in Dahl R *versus *S rat kidney cortex on normal and high salt diet

**DOI:** 10.1186/1755-7682-2-5

**Published:** 2009-03-13

**Authors:** Marlene F Shehata

**Affiliations:** 1 Department of Cellular and Molecular Medicine, Faculty of Medicine, University of Ottawa, K1Z 8M5, Ottawa, ON, Canada

## Abstract

**Aims/hypothesis:**

The α subunit of the amiloride-sensitive epithelial sodium channel (α ENaC) is critical for the expression of functional channels. In humans and rats, non functional alternatively spliced forms of α ENaC have been proposed to act as negative regulatory components for ENaC. The purpose of this study was to examine the presence and consequently investigate the mRNA expression levels of alternatively spliced forms of α ENaC in kidney cortex of Dahl salt-resistant rats (R) *versus *Dahl salt-sensitive rats (S) on high salt and normal diets.

**Methods:**

Using quantitative RT-PCR strategy, we examined the mRNA expression levels of previously reported α ENaC-a and -b alternatively spliced forms in kidney cortex of Dahl S and R rats on normal and four-week high salt diet and compared their corresponding abundance to wildtype α ENaC mRNA levels. We identified 2 novel non-coding C-terminus spliced forms and examined their mRNA expression in Dahl R *versus *S rat kidney cortex. We also tested the presence of five previously reported lung-specific α ENaC spliced forms in Dahl rat kidney cortex (CK479583, CK475461, CK364785, CK475819, and CB690980).

**Results:**

Previously reported α ENaC-a and -b alternatively spliced forms are present in Dahl rat kidney cortex and are significantly higher in Dahl R *versus *S rats (P < 0.05). Four-week high salt diet significantly increases α ENaC-b (P < 0.05), but not α ENaC-a transcript abundance in Dahl R, but not S rats. Two non-coding α ENaC spliced forms -c and -d are newly identified in the present study, whose levels are comparable in Dahl R and S rats. Compared to α ENaC-wt, α ENaC-a, -c and -d are low abundance transcripts (4 +/- 2, 110 +/- 20, and 10 +/- 2 fold less respectively), in contrast to α ENaC-b abundance that exceeds α ENaC-wt by 32 +/- 3 fold. We could not identify any of the five previously reported lung-specific α ENaC spliced forms (CK479583, CK475461, CK364785, CK475819, and CB690980) in Dahl rat kidney cortex.

**Conclusion/interpretation:**

α ENaC alternative splicing might regulate α ENaC by the formation of coding RNA species (α ENaC-a and -b) and non-coding RNA species (α ENaC-c and -d). α ENaC-a and -b mRNA levels are significantly higher in Dahl R *versus *S rats. Additionally, α ENaC-b is a salt-sensitive transcript whose levels are significantly higher 4-weeks post high salt diet compared to normal salt diet in Dahl R rats. Among the four α ENaC transcripts (-a, -b, -c and -d), α ENaC-b is a predominant transcript that exceeds α ENaC-wt abundance by ~32 fold. α ENaC-a and -b spliced forms, particularly, α ENaC-b, might potentially act as dominant negative proteins for ENaC activity, thereby rescuing Dahl R rats from developing salt-sensitive hypertension on high salt diet. On the other hand, non-coding α ENaC-c and -d might assist alternative splicing, facilitate RNA processing, or regulate α ENaC as well as each other.

## Introduction

The amiloride-sensitive epithelial sodium channel (ENaC) plays a key role in active sodium reabsorption by epithelia throughout the body, including kidney tubules, the lung, the distal colon, sweat and salivary glands, and the brain [1]. ENaC has been purified from the kidney as a large protein complex [2-4]. Molecular cloning studies indicate that ENaC consists of at least three subunits denoted by α, β, & γ, each of which possesses two transmembrane domains, a large extracellular loop, a cytoplasmic C-terminus and N-terminus domains [5,6,6]. α ENaC subunit is critical to the formation of ion conductive membrane pore, whereas β & γ ENaC subunits are required for maximal channel activity. α ENaC knockout in mice was lethal within 40 h of birth due to failure of pulmonary fluid clearance, clearly demonstrating the pivotal role of α ENaC in forming a functional Na^+ ^channel complex in vivo [7]. Moreover, decreased α ENaC expression (without necessarily knocking out α ENaC) predisposes animals to a respiratory distress syndrome [8], unlike the β and γ subunits that have only minor effects on lung fluid clearance.

ENaC activity is twice in kidneys of Dahl S *versus *R rats, possibly explaining the salt-sensitivity seen in Dahl S on high salt diet [9,10]. The reasons for this variability in ENaC activity in Dahl S *versus *R rats are poorly understood at present. We have screened ENaC α, β, and γ complete coding sequences, exon-intron junctions, 5' and 3'flanking regions in Dahl S *versus *R rats and indeed there were no genetic differences between both strains in the above sequenced regions [11]. Alternative splicing of pre-mRNA represents a widespread mechanism for increasing variability of eukaryotic gene expression and has been associated with human pathologies, such as cancer, Alzheimer's, amyotrophic lateral sclerosis, ataxia telangiectasia, cystic fibrosis, and senescence [12,13]. Alternatively spliced forms of α ENaC have been described in rats [14-17], humans [14-17], mice [14-17] and chicken [14-17]. Non functional α ENaC alternatively spliced forms have been proposed to serve as negative regulatory components for ENaC activity in humans [16]. An example of the dominant negative role played by alternatively spliced forms on full length forms can be seen in the inhibitor of apoptosis protein family. The inhibitor of apoptosis protein family was shown to be expressed at mRNA levels that were 2–3% of the levels of the full-length transcript, yet it encodes a protein that accumulates 50-fold higher levels than full-length and this accumulated protein competes with full-length form for activity [18]. The above facts have prompted us to examine the existence and consequently the mRNA expression levels of α ENaC alternatively spliced forms -a and -b in Dahl R *versus *S rats on normal and 4 week-high salt diet, and to test the presence of five previously reported lung-specific α ENaC spliced forms in Dahl rat kidney cortex. During the course of the present studies, we identified and characterized two novel non-coding C-terminus spliced forms in Dahl rat kidney cortex, and consequently determined their corresponding abundance in Dahl rats on normal and four-week high salt diet.

## Methods

### Animals

Male Dahl S and R rats (n = 24), 3–4 wks of age, were obtained from Harlan Sprague Dawley (Indianapolis, IN) and handled as previously described [19]. The rats were divided into 4 groups (6 rats/group) by placing them on either regular (120 μmol Na^+^/g) or high-salt (1,370 μmol Na^+^/g, Teklad; Madison, WI) diet for four wks. After 4 wks, blood pressure was measured invasively by intraarterial catheter and the average mean arterial pressure was estimated to be 192 ± 10 for S, and 125 ± 5 for R rats (P < 0.05). The animals were then killed by decapitation and kidney tissues were removed and placed in cold methylbutane and then on dry ice. Tissues were preserved at -80°C for later RNA isolation. All experiments were carried out in accordance with the guidelines of the University of Ottawa Animal Care Committee for the care and use of laboratory animals.

### RNA isolation

Approximately 100 mg of kidney cortex was cut out microscopically and homogenized with polytron. Total RNA was isolated using Trizol reagent (Invitrogen) according to the manufacturer's protocol. Isolated RNA was subjected to DNAse treatment for removing potential genomic DNA contamination using DNase treatment kit (Ambion). Total RNA was quantified by UV light absorbance at 260 nm by a spectrophotometer. The OD ratio of 260/280 nm was within the range of 2.0 ± 0.1 in all samples.

### Reverse transcription (RT)

5 μg of RNA were transcribed to cDNA in a total of 20 μl reaction volume. Briefly, a mixture of 1 μl (500 ng) oligo (dT)_12–18 _primer), 1 μl 10 mM deoxyribonucleotides (dNTP) mix (final conc. 0.5 mM) and water were added to the calculated volume of RNA to make 12 μl. The mixture was heated to 65°C for 5 minutes, then quickly chilled on ice, and briefly centrifuged. 4 μl 5 × first strand buffer, 2 μl 1 M DTT, 1 μl RNAse OUT Recombinant Ribonuclease Inhibitor (40 units) and 1 μl of Superscript II RNase H-Reverse Transcriptase (200 units) (Invitrogen, Burlington, ON) were added to the tube. The final mixture (20 μl) was incubated in a water bath at 42°C for 50 minutes. The reaction was inactivated at 70°C for 15 min. First strand cDNA thus obtained was cooled in ice and then stored at -20°C until PCR was performed.

### Primer design

Primers were designed specifically to amplify the α ENaC-a and -b alternatively spliced forms by including the nucleotide deletions (23 bp in α ENaC-a sense primer, and 79 bp in α ENaC-b reverse primer). The primers used to amplify α ENaC-a, -b, -c, -d and the previously reported lung spliced forms (CK479583, CK475461, CK364785, CK475819, and CB690980) are mentioned in Table 1. For α ENaC -a and -b, we used the sequences previously reported [15]; whereas for the lung spliced forms we used the sequences retrieved from the . The accession numbers for the five previously reported lung spliced forms are CK479583, CK475461, CK364785, CK475819, and CB690980. For α ENaC-c and -d, 2 different primer pairs were used to confirm the newly identified spliced forms.

**Table 1:** Primers used for reverse transcription polymerase chain reaction and expected sizes of cDNA fragments.

**Accession ID**	**Amplicon Size**	**Primers used**
α ENaC-wt	325 bp	Sense: 5'-TCATGCTGCTACGCCGGTTCC-3'

		Antisense:5'-TCCATCAGTTTACAAGGGAG-3'

α ENaC a	266 bp	Sense: 5'-CAGAGCTCCTGGGGGCGCCTT-3'

		Antisense: 5'-TCCTTCTGTCACGATGGTCA-3'

α ENaC-b	278 bp	Sense: 5'-GGATGATGGTGGCTTCAACT-3'

		Antisense: 5'-ACACCCAGGAGCTCTGCTTT-3'

α ENaC-c	59 bp	Sense: 5'-GCTCCTGGGTGTGATCAACTA-3'

		Antisense: 5'-AAAGAAGGTGGTGGGGATGT-3'

	59 bp	Sense: 5'-GGATGATGGTGGCTTCAACT-3'

		Antisense: 5'-AAAGAAGGTGGTGGGGATGT-3'

α ENaC-d	99 bp	Sense: 5'-CCACGCGTCCGAAGAAAC-3'

		Antisense:5'-AAAGAAGGTGGTGGGGATGT-3'

	99 bp	Sense: 5'-CCACGCGTCCGAAGAAACTG-3'

		Antisense:5'-TAAAGAAGGTGGTGGGGATG-5'

CK479583	767 bp	Sense: 5'-GCCCCAGCAGGGCATGACCC-3'

		Antisense: 5'-CTAGAGTAGCATAGGCAGGT-3'

CK475461	814 bp	Sense: 5'-TCCAAGTATACACAGCAGGT-3'

		Antisense: 5'-TACAGGAGTCAGGCTGTGAG-3'

CK364785	764 bp	Sense: 5'-GGGGCTATTGCTATTATAAA-3'

		Antisense:5'-CCACACAGAGGGCTCGGCGGG-3'

CK475819	748 bp	Sense: 5'-GATTCCCGGGATCTTGGA-3'

		Antisense: 5'-AGGCTGACCATCGTGACAG-3'

CB690980	376 bp	Sense: 5'-CCACGCGTCCGAAGAAAC-3'

		Antisense: 5'-AAAGAAGGTGGTGGGGATGT-3'

Pgk	263 bp	Sense: 5'-GCTGCAGAACTCAAATCTCT-3'

		Antisense:5'-TGTGTGCAGTCCCAAAAGCA-3'

### Cloning

α ENaC-a, -b, -c and -d were cloned using DH5 α competent cells, and TOPO TA cloning kit^® ^(Invitrogen, ON, Canada). The resulting clones were sequenced using ABI prism 3100 (PE Applied Biosystems, Foster City, CA). Sequencing was performed using the DYEnamic ET Terminator kit according to the instructions provided by the manufacturer (PE Applied Biosystems, Foster City, CA). Sequencing products were purified (DyeEx 2.0 spin kit columns; Qiagen Canada, Mississauga, ON, Canada). The newly identified α ENaC-c and -d spliced forms were examined using TRANSEQ^® ^computer program for translation in silico .

### Quantitative Real-time PCR

Real-time PCR amplifications were performed with a Roche Light Cycler using Fast Start DNA Master SYBR Green I (Roche Diagnostics, Montreal, QC). 2 μL of 1:10 diluted RT product from kidney cortex were used for the PCR reaction in a 20 μl reaction using the previously described primers for each splice variant. The real-time PCR conditions were as follows: first, 95°C for 10 min to activate Taq polymerase, followed by 45 cycles of denaturation at 95°C for 5 sec, annealing for 10 sec at 62° for PGK and α ENaC-wt; 65°C for α ENaC-a, -c and -d and 67°C for α ENaC-b. An extension step was performed at 72°C and the extension time was determined by the formula of amplicon length/25 sec. The specificity of real-time PCR products was documented with a melting curve analysis. In addition, a high resolution gel electrophoresis was performed which resulted in the amplification of a single product of the appropriate size (Table 1). Real-time RT-PCR analysis was performed in triplicate.

Plasmids containing the cDNAs of α ENaC-wt, -a, -b, -c and -d transcripts were used to generate external standard curves. α ENaC subunit full length cDNA plasmids was a gift from Dr. Bernard C. Rossier, Univ. of Lausanne, Switzerland. All of the construct concentrations were quantified by absorbance at 260 nm. Serial 10-fold dilution (e.g. 100 pg, 10 pg, 1 pg, 0.1 pg, 0.01 pg, 0.001 pg etc.) of each plasmid clones were used to generate an external standard individually by using same condition of real-time PCR described above for each gene. Expression was normalized to PGK levels as an endogenous reference. Normalization was achieved by dividing the amount of cDNA of each ENaC transcript by the PGK quantity. Real-time PCR efficiencies were calculated according to the equation E = 10^[-1/slope]^: for α ENaC-wt: 1.97; -a: 2.00, -b: 1.99, -c: 1.98, -d: 1.95 and PGK: 1.94.

### Examining in silico translation for α ENaC-b, -c and -d

Using Transeq^® ^computer software, α ENaC-b, -c and -d were examined for translation in silico. Transeq^® ^free web tool can translate any nucleic acid sequence to the corresponding peptide sequence in any of the three forward and three reverse sense frames.

### Statistical Analysis

Differences between the expression levels of α ENaC-wt and alternatively spliced forms were analyzed in Dahl R *versus *S rats on normal and high salt diet using two-way ANOVA (SigmaSTAT^®^). The statistical test was two sided to identify strain and dietary differences, the data were expressed as means and ranges, and differences of *P *values of less than 0.05 were considered to be significant.

## Results

### Presence of α ENaC-a, -b, -c and -d in Dahl rat kidney cortex on normal and high salt diet

**Figure 1:**
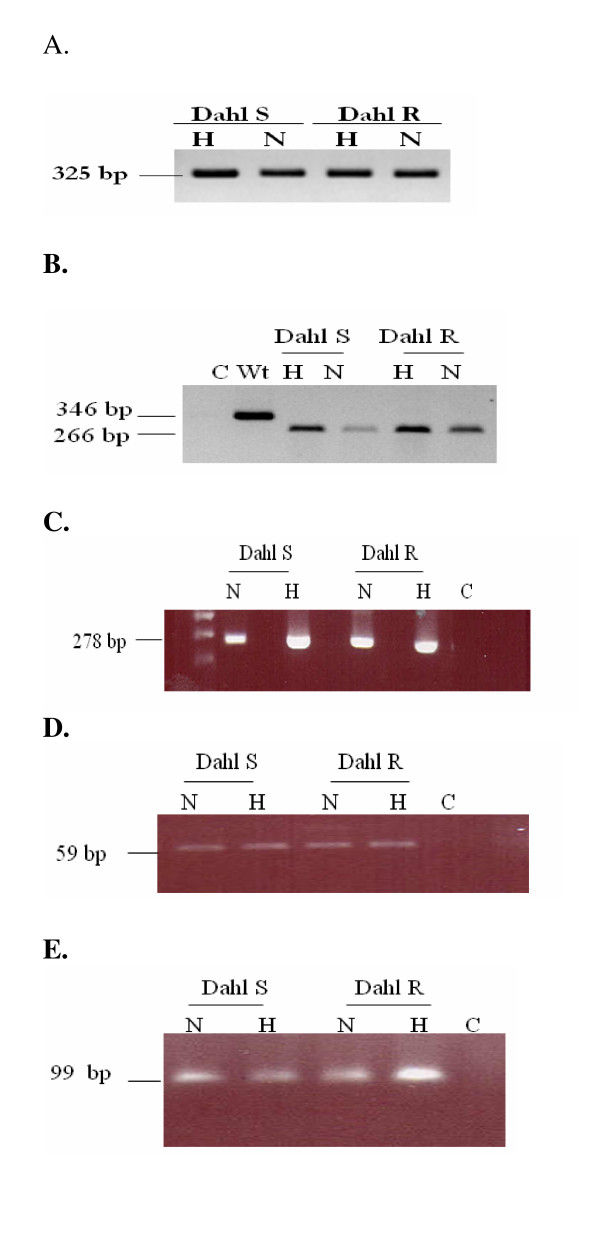
**PCR analysis of α ENaC-a, -b, -c and -d variants *versus *α ENaC-wt in kidney cortex of Dahl S *versus *R rats on normal and high salt diet**. Figure 1A) Specific sense and antisense primers were used for amplifying α ENaC-wt. α ENaC-wt fragment of 325 bp was amplified from kidney cortex of Dahl S and R rats fed normal and high salt diet. Figure 1B) Specific sense primers (missing the 23 bp unique to α ENaC-a) were utilized to amplify α ENaC-a. α ENaC-a fragment of 266 bp was amplified from kidney cortex of Dahl S and R rats fed normal and high salt diets. For a negative control, water without a cDNA template was used and for a positive control, α ENaC-wt primers were utilized to amplify α ENaC-wt fragment of 346 bp. Figure 1C) Specific antisense primers (missing the 79 bp unique to α ENaC-b) were utilized. α ENaC-b fragment of 278 bp was amplified from kidney cortex of Dahl S and R rats fed normal and high salt diet. Figure 1D) Specific primers for α ENaC-c were used to amplify a fragment of 59 bp. α ENaC-c was amplified from kidney cortex of Dahl S and R rats fed normal and high salt diet. Figure 1E) Specific primers for α ENaC-d were used to amplify a fragment of 99 bp. α ENaC-d was amplified from kidney cortex of Dahl S and R rats fed normal and high salt diet. *C (Control)*: water without cDNA template; *wt*: wildtype α ENaC primers; *H*: High salt diet; *N*: Normal salt diet. The sizes of the expected PCR products are indicated.

Amplification of α ENaC-a, -b, -c and -d was done in Dahl S and R rat kidney cortex on normal and high salt diet (Figure 1A, B, C, D). The three alternatively spliced α ENaC-a, -b, -c forms share a common 5' donor splice site (GCTCCTGGG). PCR signals for α ENaC-a, -c and -d were lower than those for α ENaC-wt. In contrast, α ENaC-b signal was higher than α ENaC-wt.

### Screening Dahl rat kidney cortex for the previously reported lung spliced forms and identification of α ENaC-c and -d spliced forms

**Figure 2:**
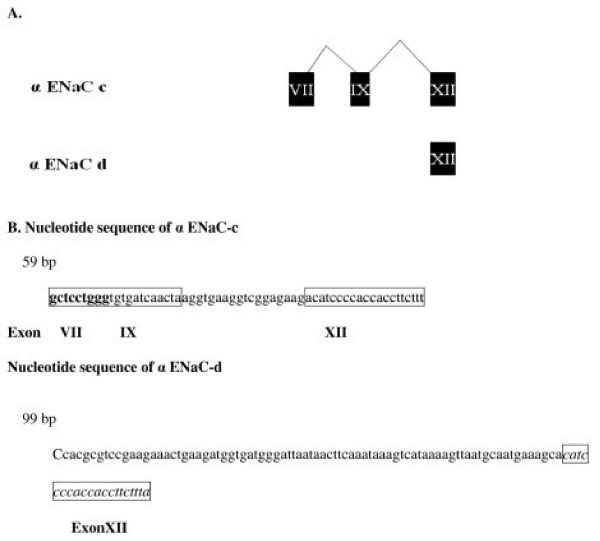
**Schematic representation of novel α ENaC alternatively spliced forms (-c and -d)**. A) A schematic illustration of the mRNA sequences of α ENaC-c and -d forms. α ENaC-c and -d are small non-coding RNA. α ENaC-c is composed of portions of exon VII, IX and XII, while that of α ENaC-d is formed of a portion of exon XII and an intronic fragment. B) The genomic sequence of α ENaC-c and -d forms. α ENaC-c share the same splicing site (CCTGGG) with α ENaC-a and -b spliced forms, which is located within exon VII. α ENaC-c and -d are made up of 59 and 99 bases respectively.

Using PCR primers to amplify the 5 previously reported spliced forms; we did not find any in Dahl rat kidney cortex (Table 1). However, we obtained and characterized 2 novel C-terminus spliced forms from Dahl rat kidney cortex. The first novel spliced form, referred to as α ENaC-c is a 59 bp RNA that was confirmed by complete nucleotide sequencing, while the second spliced form, referred to as α ENaC-d is a 99 bp RNA that was confirmed by complete nucleotide sequencing (Figure 2).

To confirm that α ENaC-c and -d are not cloning artifacts, we conducted PCR analysis using selective primers that would amplify the 59 bp and 99 bp variants, and we cloned and subsequently sequenced the resulting amplicons. Sequencing results showed that α ENaC-c is the shortest isoform, made up of 9 nt of exon 7 (GCTCCTGGG), 12 nt of exon 9 (TGTGATCAACT) and 20 nt of exon 12 (ACATCCCCACCACCTTCTTT). α ENaC-d is the second shortest variant and included 20 nt of exon 12.

### Examining α ENaC-b, -c and -d translation in silico

α ENaC-b is translatable as predicted by Transeq^® ^web tool, and as previously reported [15]. Both newly identified spliced forms α ENaC-c and -d were non-coding RNA. The peptide sequences as predicted by Transeq^® ^computer software were interrupted with multiple stop codons in each of the three forward and three reverse sense frames, and this in turn hinders the translation of α ENaC-c and -d into a polypeptide sequence.

### Comparison of mRNA expression levels of α ENaC -a, -b, -c and -d to α ENaC-wt using quantitative RT-PCR strategy

Using PCR primers that were designed to amplify α ENaC -a and -b, we amplified and subsequently cloned 266 bp and 278 bp of alternatively spliced forms α ENaC-a and -b respectively. We confirmed the deletions in α ENaC-a and -b by nucleotide sequencing of the resulting amplicons.

**Figure 3:**
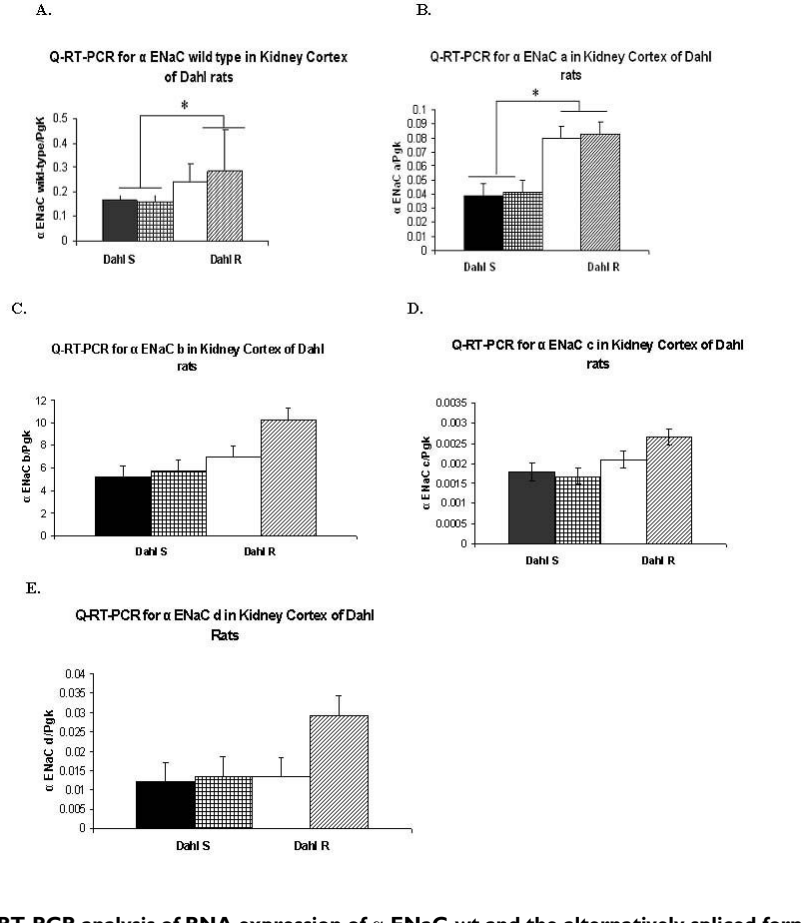
**Quantitative RT-PCR analysis of RNA expression of α ENaC-wt and the alternatively spliced forms -a, -b, -c and -d in kidney cortex of Dahl S and R rats fed normal and high salt diet**. Figure 3A) Specific primers for α ENaC-wt were used to amplify cDNAs from kidney cortex of Dahl S and R rats on normal and high salt diet. A significant increase (P < 0.05) in α ENaC-wt expression was observed in Dahl R *versus *S rats. No significant dietary impact on the expression levels of α ENaC-wt was noticed in Dahl S or R rats. Figure 3B) Specific primers unique to α ENaC-a were used to amplify cDNAs from kidney cortex of Dahl S and R rats on normal and high salt diet. A significant increase (P < 0.05) in α ENaC-a expression was observed in Dahl R *versus *S rats. No significant dietary impact on the expression levels of α ENaC-a was noticed in Dahl S or R rats. Figure 3C) Specific primers unique to α ENaC-b were used to amplify cDNAs from kidney cortex of Dahl S and R rats on normal and high salt diet. A significant increase (P < 0.05) in α ENaC-b expression was observed in Dahl R *versus *S rats. Additionally, a significant increase in the expression levels of α ENaC-b was observed on high *versus *normal salt diet in Dahl R, but not S rats. Figure 3D) Specific primers unique to α ENaC-c were used to amplify cDNAs from kidney cortex of Dahl S and R rats on normal and high salt diet. Only a slight, insignificant increase in α ENaC-c expression was observed in Dahl R *versus *S rats. No significant dietary impact on the expression levels of α ENaC-c was noticed in Dahl S or R rats. Figure 3E) Specific primers unique to α ENaC-d were used to amplify cDNAs from kidney cortex of Dahl S and R rats on normal and high salt diet. Only a slight, insignificant increase in α ENaC-a expression was observed in Dahl R *versus *S rats. α ENaC-a, -c and -d are lower abundant transcripts. Only α ENaC-b exceeded α ENaC-wt expression by ~32 fold. Bars represent mean +/- SEM, *: denotes P < 0.05 between Dahl R and Dahl S, #: denotes P < 0.05 in transcript abundance on normal and 4 week high salt diet in Dahl R, Dahl R: Dahl salt-resistant, Dahl S: Dahl salt-sensitive. All α ENaC transcript levels were normalized against the house keeping gene PgK. N = 6 rats/group and the results are the average of 3 independent experiments.

α ENaC spliced forms -a, and -b were significantly higher in Dahl R *versus *S rats (P < 0.05), a pattern similar to α ENaC-wt mRNA expression (Figure 3). A statistically significant increase of P < 0.05 was seen only in Dahl R rats for α ENaC-b spliced form on four-week high salt diet. α ENaC-c and -d expression levels were comparable in Dahl S and R rats, with no significant dietary impact on their corresponding mRNA expression levels.

α ENaC-a, -c and -d were respectively 4 +/- 2 fold, 110 +/- 20 fold, and 10 +/- 2 fold lower in abundance than α ENaC-wt (Figure 3), while α ENaC-b transcript was 32 +/- 3 fold higher than α ENaC-wt.

## Discussion

The present study demonstrates the following: α ENaC-a, -b, -c and -d are expressed in the kidney cortex of Dahl S and R rats on high and normal salt diet, and the mRNA expression levels of α ENaC alternatively spliced forms – α ENaC-a and -b – are significantly higher (P < 0.05) in Dahl R *versus *S rats. Compared to normal salt diet, four-week high salt diet caused only a slight, insignificant elevation in α ENaC-a, whereas a moderate increase in the abundance of α ENaC-b (P < 0.05) in Dahl R rats was determined. Two novel non-coding spliced forms referred to as α ENaC-c and -d were characterized, cloned and sequenced from Dahl rat kidney cortex. α ENaC-c and -d mRNA abundance were insignificantly higher (up to 1.6 fold) in Dahl R *versus *S rats. None of the previously reported lung spliced forms (CK479583, CK475461, CK364785, CK475819, and CB690980) were identified in kidney cortex of Dahl S, nor R rats on normal or four- week high salt diet. Comparing α ENaC alternatively spliced forms to α ENaC-wt transcript abundance, only α ENaC-b was 32 +/- 3 folds higher than α ENaC-wt, while α ENaC-a, -c and -d were respectively 4 +/- 2 fold, 110 +/- 20 fold, and 10 +/- 2 fold lower in abundance than α ENaC-wt.

We chose to study α ENaC alternatively spliced forms' abundance four weeks post high salt diet in kidney cortex and not the medulla of Dahl S and R rats, because of the significant strain differences already reported in α ENaC-wt expression levels. Kidney medulla α ENaC-wt mRNA was not affected by either diet or strain at any time point (two or four-week high salt diet) [20], whereas kidney cortex α ENaC-wt mRNA was significantly higher in Dahl R *versus *S rats kidney after four, but not two weeks of high salt diet [20].

Alternative splicing is a major contributor to the structural and functional diversity of ion channels such as the Na^+^, K^+^, and Ca^++ ^channels [21-25]. Formation of alternatively spliced forms of α ENaC have been previously reported in four species: humans [14-17], rats [14-17], mice [14-17] and chicken [14-17]. In rats, two alternatively spliced forms (α ENaC-a and -b) of the α ENaC subunit are currently published [14-17]. α ENaC -a and -b are identified in the rat kidney and tongue taste tissues [14-17]. Both α ENaC -a and -b encode for shorter proteins that lack the second transmembrane domain M2. α ENaC-a has been expressed in HEK293 and CV1 cells [14-17], while α ENaC-b translation *in vitro *is yet to be determined. When α ENaC-a is expressed in *Xenopus *oocytes, loss of channel function was reported suggesting that α ENaC-a polypeptide might hinder α ENaC-wt expression and/or function by direct physical interaction, or by disrupting channel assembly in vivo because of the critical involvement of the missing transmembrane domain in creating functional channels [14-17].

Our screening study yielded preliminary evidence that suggested the generation of additional spliced forms in kidney cortex (α ENaC-c and -d) besides the previously reported spliced forms (α ENaC-a and -b). α ENaC-a, -b, and -c spliced forms share the same 5' donor splice site (GCTCCTGGG) with the human α ENaC +22 variant [14-17] and the chicken 3399 bp variant [14-17]; suggesting that this 5'donor splice site is preferably utilized by various species to generate spliced forms of the α ENaC subunit [14-17]. There was an insignificant elevation (up to 2 fold) in α ENaC-c and -d mRNA in Dahl R *versus *S rats which might have possibly reached significance by increasing the number of rats screened (n > 6). When α ENaC-c and -d were examined for translation in silico using Transeq^®^, they appeared to be non-coding. It is not certain at this point whether α ENaC-c and -d, are the result of aberrant splicing or the result of regulated alternative splicing.

Until very recently there were no reports on the regulation of ENaC in Dahl S and R rat models. Then, Aoi et al. reported an abnormal increase in α ENaC-wt mRNA (2.5-fold) in the kidneys of Dahl S, but not R rats (α ENaC mRNA is suppressed in Dahl R rats) on high *versus *regular salt intake for 4 weeks [26,27]. It is worth mentioning that the sequences of the forward and reverse primers used by Aoi and co-authors [26,27] are common among α ENaC-wt, α ENaC-a and -b spliced forms, and the altered levels reported for α ENaC should be carefully interpreted (because the primers would amplify both the major transcript and the two alternatively spliced forms, and hence cause false elevations of full-length α ENaC mRNA). Our results, on the other hand, using specific α ENaC-wt primers showed no significant changes in α ENaC-wt mRNA concentration in response to salt in Dahl S and R rats, but a constitutive increase in α ENaC-wt mRNA concentration was witnessed in Dahl R *versus *S rats.

Indeed, the increased levels of expression of α ENaC-a and -b transcripts in Dahl R *versus *S rats might be indicative of a putative regulatory effect on α ENaC expression/function. In conclusion, α ENaC alternative splicing might play a role in ENaC regulation by the resulting coding (α ENaC-a and -b) and non-coding (α ENaC-c and -d) alternatively spliced forms. α ENaC-a and -b coding transcripts allow the expression of shortened, but partially functional proteins. The highly abundant α ENaC-b transcript might possibly play a role as a dominant negative element in Dahl R rats rescuing the α ENaC from overactivity in high salt environment, either by direct interaction with α ENaC or by affecting α ENaC proper assembly and the formation of functional channels. The non-coding newly identified α ENaC-c and -d alternatively spliced forms might act as potential regulators for the RNA machinery possibly by facilitating RNA processing, assisting alternative splicing, regulating α ENaC as well as each other or finally they might be an indicative of the cells' potential to tolerate a splicing process which generates frequent splicing errors. It is worth mentioning that at the time of submission of the current manuscript, preliminary evidence of the translation of α ENaC-b *in vitro *and its putative dominant negative effect on α ENaC-wt expression has been demonstrated [28,29].

## Competing interests

The author declares no competing interests.
